# Temporal Expression of a Master Regulator Drives Synchronous Sporulation in Budding Yeast

**DOI:** 10.1534/g3.116.034983

**Published:** 2016-09-06

**Authors:** Minghao Chia, Folkert J. van Werven

**Affiliations:** Cell Fate and Gene Regulation Laboratory, The Francis Crick Institute, London WC2A 3LY, UK

**Keywords:** gametogenesis, sporulation, synchrony, budding yeast, DNA replication, meiotic divisions, *IME1*, *IME4*, temporal

## Abstract

Yeast cells enter and undergo gametogenesis relatively asynchronously, making it technically challenging to perform stage-specific genomic and biochemical analyses. Cell-to-cell variation in the expression of the master regulator of entry into sporulation, *IME1*, has been implicated to be the underlying cause of asynchronous sporulation. Here, we find that timing of *IME1* expression is of critical importance for inducing cells to undergo sporulation synchronously. When we force expression of *IME1* from an inducible promoter in cells incubated in sporulation medium for 2 hr, the vast majority of cells exhibit synchrony during premeiotic DNA replication and meiotic divisions. Inducing *IME1* expression too early or too late affects the synchrony of sporulation. Surprisingly, our approach for synchronous sporulation does not require growth in acetate-containing medium, but can be achieved in cells grown in rich medium until saturation. Our system requires solely *IME1*, because the expression of the *N*6-methyladenosine methyltransferase *IME4*, another key regulator of early sporulation, is controlled by *IME1* itself. The approach described here can be combined easily with other stage-specific synchronization methods, and thereby applied to study specific stages of sporulation, or the complete sporulation program.

Gametogenesis—the process of gamete formation—is an essential cell differentiation program for all sexually reproducing organisms. During gametogenesis, diploid cells undergo a single round of DNA replication, followed by double-strand break formation, homologous recombination, and two consecutive nuclear divisions called meiosis I and II, to generate progeny containing half the number of chromosomes of the diploid parent cell (Marston and Amon 2004). The products of meiotic divisions are subsequently packaged to form gametes or spores in yeasts.

In budding yeast, initiation of gametogenesis, or sporulation, is controlled by a single master regulatory transcription factor called inducer of meiosis 1 (*IME1*) (Kassir *et al.* 1988). Ime1 activates transcription of the early genes during sporulation (Honigberg and Purnapatre 2003). Extrinsic and intrinsic signals control *IME1* expression via the *IME1* promoter (van Werven and Amon 2011). For efficient *IME1* induction, glucose and nitrogen needs to be absent from the growth medium, and cells need to express both mating types (*MAT*a and *MAT*α). Another gene important for entry into gametogenesis is *IME4*, which encodes an enzyme that methylates the adenosine base of mRNAs to form *N*-6-methyladenosine (m6a) (Shah and Clancy 1992; Clancy *et al.* 2002). Previous work indicated that Ime4 promotes the accumulation of *IME1* transcripts, suggesting that there is positive regulation between the two genes during entry into sporulation (Shah and Clancy 1992).

Yeast cells undergo gametogenesis relatively asynchronously, making it challenging to perform stage-specific analyses using whole cell population based assays. Nachman *et al.* (2007) showed that cell-to-cell variability in *IME1* levels causes variation in timing of meiotic events in a population of cells. In contrast, nutritional history, cell cycle stage, or cell size did not affect timing of meiotic divisions (Nachman *et al.* 2007). We reveal new insights into *IME1* signaling, and describe a method to synchronize premeiotic DNA replication and meiotic divisions. First, we demonstrate that timed expression of *IME1*, but not *IME1* expression by itself, is sufficient to drive synchronous sporulation. In addition, we find that *IME1* expression regulates *IME4* expression, explaining our observation that cells readily enter sporulation highly synchronously when *IME1* is induced alone. Surprisingly, our system does not require growth in acetate-containing medium, but synchronous sporulation can be induced directly in cells grown in rich medium until saturation before shifting to sporulation medium. Finally, we show that the approach described here can be combined with other stage-specific synchronization methods to study specific stages of sporulation, or the complete sporulation program.

## Materials and Methods

### Yeast strains

All yeast strains used in this paper were derived from the sporulation proficient SK1 strain background, and genotypes are listed in Supplemental Material, Table S1. The *CUP1* promoter fusion with *IME1* (*pCUP-IME1*) strain was made as follows: a region of the *pFA6a-KanMX6-pCUP1A-3HA* plasmid was amplified using the primers 5′-GCATTGATATTTTCAAACTTATATAATTAATAATAATTAATAGCGCTTAGTTTAAAGAAgaattcgagctcgtttaaac-3′ and 5′-GAAACCATCTTCTAAGGCAGCGTGAAGTTTTCCATG CATATCCGCTTGCATgcactgagcagcgtaatctg-3′. Uppercase letters refer to *IME1*-specific sequences, while lowercase letters refer to the sequences for amplifying the *CUP1* promoter with N-terminal 3HA tags (Longtine *et al.* 1998). For the untagged version of *pCUP-IME1*, the *pFA6a-KanMX6-pCUP1A-3HA* plasmid was amplified using the primers 5′-GCATTGATATTTTCAAACTTATATAATTAATAATAATTAATAGCGCTTAGTTTAAAGAAgaattcgagctcgtttaaac-3′ and 5′-CTAAGGCAGCGTGAAGTTTTCCATGCATATCCGCTTGCATtttatgtgatgattgattgattg-3′. The strains were generated by a one-step promoter replacement protocol as described by Longtine *et al.* (1998). Subsequently, the haploid transformants were backcrossed, from which homozygous diploid cells were generated.

### Growth and conditions for synchronous sporulation

Cells were grown in YPD [1% yeast extract, 2% peptone, 2% glucose, and supplemented with tryptophan (9.6 mg/l), uracil (2.4 mg/l), and adenine (1.2 mg/l)] and grown to exponential phase (OD_600_ < 2.0) at 30° and 300 rpm. While developing the protocol, we found that supplemented tryptophan can be left out from the YPD. For optimal aeration, the ratio of the total volume of the flask to the volume of medium was at least 10:1. Approximately 0.05 OD of exponentially growing yeast were inoculated into a new flask containing reduced glucose YPD [1% yeast extract, 2% peptone, 1% glucose, and supplemented with uracil (2.4 mg/l), and adenine (1.2 mg/l)]. Cultures reached OD_600_ = 11.0–12.0 after 16–18 hr, and the majority of the cells (∼90%) were single, unbudded cells as observed under a light microscope. The cells were then pelleted by centrifugation (2000 × *g*, 3 min, room temperature). The pellets were washed with sterile Mili-Q water, centrifuged again (2000 × *g*, 3 min, room temperature), and suspended in sporulation medium [1.0% (w/v) potassium acetate, supplemented with adenine/uracil (40 mg/l each), histidine/leucine/tryptophan (20 mg/l each), and 0.02% (w/v) raffinose)] at OD_600_ of 2.5. After 2 hr, copper (II) sulfate (50 µM) was added to induce expression from the *CUP1* promoter and initiate sporulation synchronously. For some experiments sporulation was induced by growing cells in acetate containing pre-sporulation medium (BYTA) followed by shifting cells to sporulation medium as described previously (Berchowitz *et al*. 2013).

### Budding index determination

Cells were grown in regular YPD [1% yeast extract, 2% peptone, 2% glucose, and supplemented with tryptophan (9.6 mg/l), uracil (2.4 mg/l), and adenine (1.2 mg/l)] and grown to exponential phase (OD_600_ < 2) at 30° and 300 rpm. Cells were transferred to new flasks (OD_600_ = 0.05) containing reduced glucose YPD [1% yeast extract, 2% peptone, 1% glucose, and supplemented with uracil (2.4 mg/l) and adenine (1.2 mg/l)] or regular YPD with 2% glucose. After 16–18 hr, budded and unbudded cells were counted under a light microscope.

### Flow cytometry analysis

Premeiotic DNA replication was monitored by flow cytometry analysis (BD LSRFortessa, BD Biosciences). Cells were pelleted by centrifugation (∼2400 × *g*, 1 min, room temperature), and fixed in 80% (v/v) ethanol for at least 60 min before further processing. Fixed cells were pelleted by centrifugation (∼2400 × *g*, 1 min), and resuspended in 50 mM Tris-HCl pH 7.5. Cells were sonicated for a few seconds before treatment with 0.2 mg/ml ribonuclease A in 50 mM Tris-HCl pH 7.5 at 37° overnight. After ribonuclease A digestion, cells were stained with 50 µg/ml propidium iodide in FACs buffer (200 mM Tris-HCl pH 7.5, 211 mM NaCl and 78 mM MgCl_2_) for 1 hr at room temperature before flow cytometry analysis. Propidium iodide stained cells were excited with a 561 nm yellow-green laser, and signals were detected using a 610/20 yellow filter. Pulse shape analysis (pulse height against pulse area) was used to exclude clumps and doublets. DNA content from single cells was estimated with a histogram of counts against pulse area. At least 50,000 cells were used for the analysis of each sample.

### Nuclei/DAPI counting

To monitor meiotic divisions by DAPI staining, cells were pelleted by centrifugation (∼2400 × *g*, 1 min, room temperature), and fixed in 80% (v/v) ethanol for at least 60 min before further processing. Subsequently, samples were pelleted by centrifugation (∼2400 × *g*, 1 min) and resuspended in PBS with DAPI (1 µg/ml). Cells were sonicated for a few seconds, and left in the dark at room temperature for at least 5 min. After DAPI staining, the proportion of cells containing one, two, three, or four DAPI masses were counted using a fluorescent microscope.

### Computing the synchrony of meiotic divisions

The synchrony of meiotic divisions for each time course experiment was approximated by fitting a linear trend line from the first time point when meiotic divisions were detected to the first time point when 75% or more of the cells completed meiotic divisions. From these analyses, we calculated the period, or time taken for 75% of the cells to complete meiotic divisions. A more synchronously dividing population would take a shorter time to complete meiotic divisions. The average values from three independent experiments, and the SEM are included in the figures. To illustrate the statistical significance we used one way ANOVA, *post hoc* multiple comparison testing, and two tailed *t*-tests where appropriate (Prism 6, Graphpad). A *p*-value ≤ 0.05 was considered significant.

### Western blotting

Levels of hemagglutinin (HA) epitope-tagged Ime1 and Ime4 were determined by western blotting using the procedures as described previously (Berchowitz *et al.* 2013). In brief, cells were pelleted by centrifugation (∼2400 × *g*, 1 min, room temperature), and resuspended in cold 5% trichloroacetic acid (TCA) for at least 10 min. The pellets were then washed with acetone, mixed with lysis buffer [50 mM Tris pH 7.5, 1 mM EDTA, 2.75 mM dithiothreitol (DTT)], and cells were broken using a mini beadbeater (BioSpec). Lysates were mixed with SDS loading buffer (187.5 mM Tris pH 6.8, 6% v/v β-mercaptoethanol, 30% v/v glycerol, 9% w/v SDS, 0.05% w/v Bromophenol Blue), and boiled for 5 min for denaturation. Proteins were separated by PAGE, and transferred onto PVDF membranes using the Mini Trans-Blot Cell (Bio-Rad). The membranes were blocked for 60 min in blocking buffer (1% w/v BSA, 1% w/v milk), before incubation with mouse anti-HA (12CA5, Sigma-Aldrich) at a 1:1000 dilution overnight at 4°. Membranes were washed in phosphate buffered saline with 0.01% Tween-20 (PBST), and incubated with anti-mouse HRP secondary antibodies (GE Healthcare) at a 1:5000 dilution. Membranes were imaged using Imagequant 600 RGB (GE Healthcare). We also monitored Hxk1 levels using an anti-hexokinase antibody (H2035, Stratech) at a 1:8000 dilution overnight at 4°. The IRDye 680RD donkey anti-rabbit secondary antibody (LI-COR) was used at a 1:15,000 dilution. Hxk1 levels were detected using an Odyssey Imager (LI-COR).

### RT-qPCR

Total RNA was treated with DNAse and purified (Macherey-Nagel); 750 ng of total RNA was used for the reverse transcription reaction using Superscript III (Life Technologies), and single-stranded cDNA were quantified by real-time PCR using SYBR green mix (Life Technologies). To measure *IME1* mRNA levels, random primers were used for the reverse transcription reaction. Since *IME4* has antisense transcription, we used an *IME4* sense-strand specific primer (5′-ATTCTGCTTGGCCTCAGCAT-3′), and an *ACT1* sense-strand specific primer (5′-TTAGAAACACTTGTGGTGAA-3′) during the reverse transcription reaction. The *IME1* and *IME4* signals were normalized to *ACT1* transcript levels. The qPCR primer sequences used for *IME1* were: 5′-CAACGCCTCCGATAATGTATATG-3′ and 5′-ACGTCGAAGGCAATTTCTAATG-3′. The qPCR primer sequences used for *IME4* were: 5′-ACCCATGCCAGAAAACTAGAGA-3′ and 5′-CGTAAATGCAATTTCCTGTCAA-3′. The qPCR primer sequences used for *ACT1* were: 5′-GTACCACCATGTTCCCAGGTATT-3′ and 5′-AGATGGACCACTTTCGTCGT-3′.

### Chromatin immunoprecipitation

Chromatin immunoprecipitation (ChIP) experiments were performed as described previously (van Werven *et al.* 2012). In short, cells were fixed with formaldehyde (1% for 20 min), which was quenched with glycine (125 mM). After breaking cells using a mini beadbeater (BioSpec), crosslinked chromatin was sheared by sonication using Bioruptor (Diagenode, six cycles of 30 sec on/off). Extracts were incubated with anti-V5 agarose beads (Sigma) for 2 hr, and beads were washed. Ume6-V5 binding was measured by real-time PCR using SYBR green mix (Life Technologies), and primers corresponding to the *IME4* promoter (5′-CGTCTTTAGGCGGCTTTTGG-3′ and 5′-ACCGATCTTCCAGAATGCCG-3′) on a 7500 Fast Real-Time PCR system (Life Technologies). The mating type locus *HMR* (5′-ACGATCCCCGTCCAAGTTATG-3′ and 5′-CTTCAAAGGAGTCTTAATTTCCCTG-3′) was used as a nonbinding control (van Werven *et al.* 2012).

### Data availability

The authors state that all data necessary for confirming the conclusions presented in the article are represented fully within the article.

## Results

### Synchronous sporulation requires specific timing of IME1 and IME4 induction

**Figure 1:**
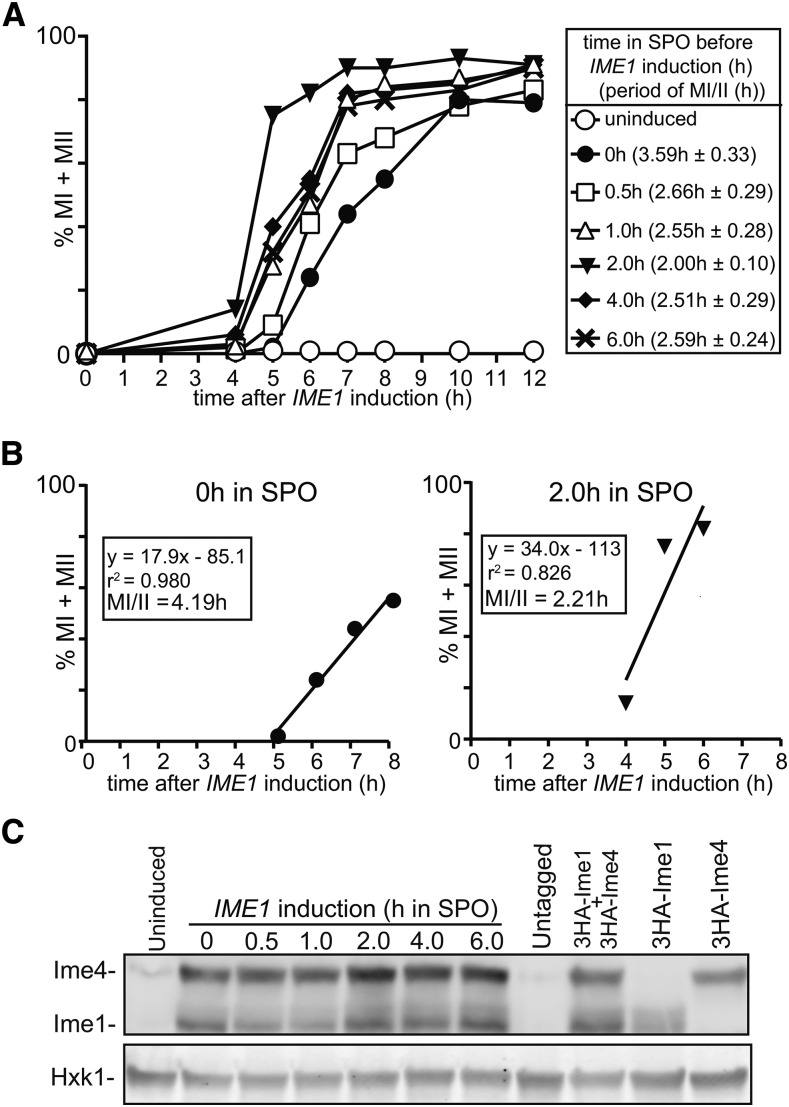
Synchronous sporulation requires specific timing of *IME1* and *IME4* induction. (A) Kinetics of meiotic divisions in diploid cells harboring *CUP1* promoter fusions with *IME1* and *IME4* (*pCUP-IME1/pCUP-IME4*) (FW1810). Cells were grown overnight in rich medium (YPD), diluted to presporulation medium (BYTA), and grown for another 16 hr. Subsequently cells were transferred to sporulation medium (SPO), and *IME1* and *IME4* were induced at 0, 0.5, 1, 2, 4, and 6 hr in SPO. Samples were collected at 4 hr after induction up to 12 hr with a 1-hr interval, fixed in ethanol, nuclei were stained with DAPI, and DAPI masses were counted. Cells that harbored two, three, or four DAPI masses were classified as cells undergoing meiosis I or meiosis II (% MI + MII, *y*-axis). For each time point, at least 200 cells were counted. The time after *IME1/IME4* induction is plotted on the *x*-axis. From each time course experiment, we also computed the time or period taken for 75% of the cells to complete meiotic divisions (see *Materials and Methods* for details). This number is displayed in brackets next to the legend, and represents the mean number of hours from three independent experiments followed by the SEM. (B) Graph to illustrate how we determined the time or period taken to complete meiotic divisions when *IME1* and *IME4* were induced at 0 or 2 hr after shifting cells to SPO medium as described in (A). A linear trend line was fitted from the first time point where meiotic divisions were detected, to the time point where 75% or more of the cells completed meiotic divisions. From the function, we calculated the period or time taken for 75% of the cells to complete meiotic divisions (MI/II). (C) Western blot showing Ime1 and Ime4 protein levels in cells described in (A). Samples were taken at 2 hr after inducing *IME1* and *IME4*. Ime1 and Ime4 levels were detected by anti-hemagglutinin (HA) antibodies. We also measured Ime1 and Ime4 in an untagged control (FW1511), and in cells that contain HA-tagged *IME1* (FW2444), or *IME4* (FW2480) alone. To control for loading, Hxk1 levels were also determined.

Whereas many of the laboratory yeast strains sporulate poorly, the sporulation-proficient strain background SK1 can undergo premeiotic DNA replication and meiosis division with a certain degree of synchrony (Cha *et al.* 2000; Stuart 2008). Nevertheless, to study specific stages of sporulation or meiosis, a highly synchronous cell population is desirable, which led to the development of different strategies to further improve the synchrony of sporulation (Wan *et al.* 2006; Carlile and Amon 2008; Berchowitz *et al.* 2013). Previous work showed that expressing *IME1* together with *IME4* from the inducible *CUP1* promoter (*CUP-IME1* and *CUP-IME4*) drives cells to undergo gametogenesis more synchronously compared to wild-type SK1 (Berchowitz *et al.* 2013). For this procedure, cells were grown in rich medium (YPD) until saturation, shifted to presporulation medium (BYTA), then transferred to sporulation medium (SPO). Subsequently, cells were incubated in SPO for 2 hr before *IME1* and *IME4* were induced with copper (II) sulfate. We speculated that the timing of *IME1* and *IME4* induction in SPO could be an important factor in regulating sporulation, since the expression of these genes are tightly regulated (Chu *et al.* 1998; Primig *et al.* 2000; van Werven and Amon 2011). To examine this, we expressed *IME1* and *IME4* at different times, and quantified the percentage of cells that completed meiotic divisions for a series of time points (Figure 1A). From these data, we estimated the synchrony of meiotic divisions by computing the time or period taken for 75% of the cells to complete meiotic divisions (see *Materials and Methods* for details) (Figure 1, A and B). The shorter the time or period, the more synchronous the meiotic divisions. We also conducted a one-way ANOVA and a *post hoc* Tukey’s test to compare the effect of expressing *IME1* and *IME4* at different times on the period taken to complete meiotic divisions. The ANOVA showed that the effect of expressing *IME1* and *IME4* at different times was statistically significant, *F*(5,12) = 3.82, *P* = 0.0265. We observed an improvement in the synchrony of meiotic divisions significantly when *IME1* and *IME4* were induced at 2 hr after shifting to SPO instead of at 0 hr (4.19 hr compared to 2.21 hr, *P* = 0.0112) (Figure 1B). Interestingly, inducing *IME1* and *IME4* either earlier or later resulted did not improve the synchrony significantly (*P* > 0.05), suggesting that there is an optimal period to induce the two master regulators. The differences in kinetics cannot be explained by Ime1 and Ime4 protein levels since they were comparable between the different samples (Figure 1C). In conclusion, our result shows that the timing of *IME1* and *IME4* induction in sporulation medium contributes to synchronous meiotic divisions.

### Cells do not require growth in acetate-containing medium prior to induction of synchronous sporulation

**Figure 2:**
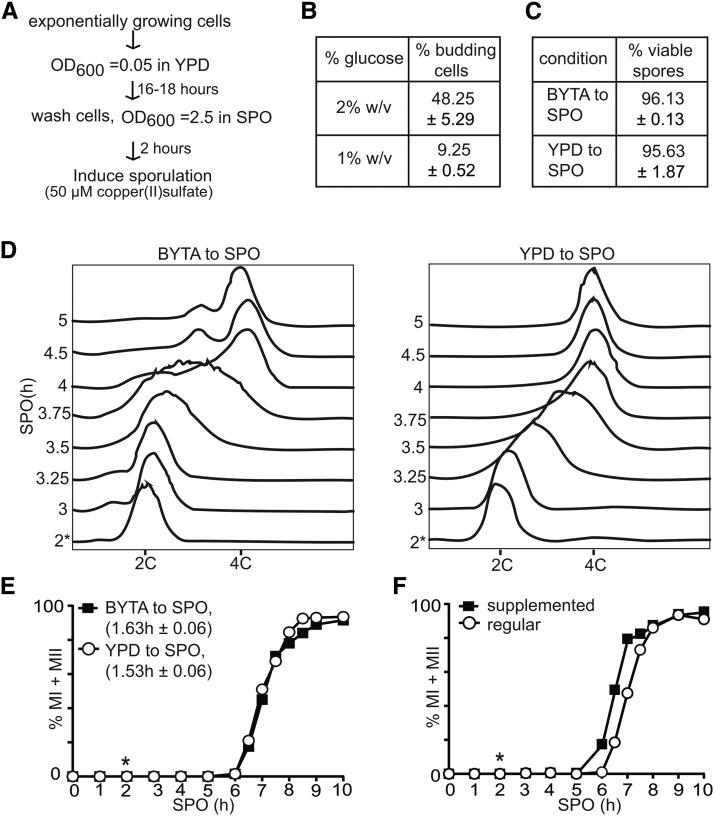
Cells do not require growth in acetate-containing medium prior to induction of synchronous sporulation. (A) Flowchart for inducing synchronous sporulation. Diploid *pCUP-IME1/pCUP-IME4* (FW1810) cells were grown to exponential phase for 6–7 hr in YPD. Cells were subsequently diluted to YPD medium with 1% glucose and grown for 16–18 hr to an OD_600_ of 11–12 to obtain mostly unbudded G_1_ cells. Cells were then pelleted by centrifugation, washed with sterile water and resuspended to a final OD_600_ of 2.5 in SPO; 50 µM copper (II) sulfate was added 2 hr after the cells were transferred to SPO to induce *IME1* and *IME4*. (B) Budding index of cells cultured for 16 hr in YPD with different glucose concentrations. The mean and SEM from three independent experiments is shown, and *n* = 400 cells were counted for each repeat. (C) Spore viability of the *pCUP-IME1*/*pCUP-IME4* strain. Cells were grown overnight in YPD, and induced to sporulate in SPO after transfer from YPD or presporulation media (BYTA). Sporulation was induced using standard protocols (BYTA to SPO), or by using the method described in (A) (YPD to SPO). Copper (II) sulfate was added 2 hr after the cells were transferred to SPO. Tetrads were collected 24 hr after *IME1* induction, dissected, and assayed for viability (*n* = 160 spores). The mean value of three independent experiments plus the SEM is shown. (D) Flow cytometry analysis of DNA content of cells cultured in either reduced glucose YPD or presporulation medium (BYTA) before shifting to SPO. Samples were taken at indicated time points, fixed, and DNA content was measured by propidium iodide staining. At least 50,000 cells were analyzed at each time point. (E) Kinetics of meiotic divisions in cells as described in (C) and (D). For determining the kinetics of meiotic divisions, samples were taken at the indicated time point, fixed, and DAPI masses were counted. Cells that harbored two, three, or four DAPI masses were classified as cells undergoing meiosis I or meiosis II (% MI + MII). For each time point, at least 200 cells were counted. We also computed the time or period taken for 75% of the cells to complete meiotic divisions (see *Materials and Methods* for details). This number is displayed in brackets next to the legend, and represents the mean number of hours followed by the SEM of three independent experiments. (F) Kinetics of meiotic divisions of the *pCUP-IME1*/*pCUP-IME4* strain as described (A) and (E), except that sporulation was induced in either regular SPO or supplemented SPO (see *Materials and Methods*). The graph displays a representative experiment from three repeated experiments. *, time of induction of *IME1*, 2 hr after the cells were transferred to SPO.

Efficient *IME1* transcription requires glucose and nitrogen starvation, and the presence of a nonfermentable carbon source in the growth medium (Kassir *et al.* 1988). To obtain high levels of *IME1* in SPO, cells are usually pregrown in acetate-containing medium. In contrast, induction of transcription from the *CUP1* promoter requires solely the presence of copper ions in the medium. If variability in the onset of meiotic divisions is dependent largely on *IME1* and *IME4* levels, then pregrowth in acetate-containing medium should be dispensable when entry into sporulation is induced from the *CUP1* promoter. Hence, we tested if the *pCUP-IME1*/*pCUP-IME4* system can induce gametogenesis synchronously when cells were pregrown in glucose containing medium (YPD), and shifted to SPO directly (Figure 2A). To ensure that all cells were arrested as unbudded cells, we grew cells in YPD with reduced glucose (1% w/v instead of 2% w/v) for 16–18 hr. Whereas the majority of cells were budding when grown in medium with standard glucose levels, the reduced glucose condition enriched for unbudded cells (∼90%) after an overnight culture (Figure 2B). Cells pregrown in YPD or BYTA also gave rise to viable spores (Figure 2C). Next, we shifted cells to SPO medium, incubated cells for 2 hr, subsequently induced *IME1* and *IME4* with copper (II) sulfate, and measured the kinetics of meiotic S-phase and meiotic divisions. We observed that cells pregrown in BYTA or YPD both completed premeiotic DNA replication in ∼60 min (Figure 2D). Remarkably, in the YPD to SPO condition, the DNA profile showed intermediate peaks for several time points (between 2C and 4C) indicating that the population of cells underwent DNA replication with a high degree of synchrony (Figure 2D). When the cells were pregrown in BYTA, these intermediate peaks were less pronounced. In addition, we also measured the rate of meiotic divisions, and found the majority of cells completed meiotic divisions within comparable periods in both conditions (Figure 2E). We conducted a two-tailed *t*-test, and found that the time taken to complete meiotic divisions under both conditions was not statistically significantly different (*P* > 0.05). Overall, our results show that synchronous DNA replication and meiotic divisions can be induced from cells precultured until saturation in nutrient-rich medium containing glucose.

We also examined whether the composition of SPO medium influences the synchrony of meiotic divisions of cells pregrown in YPD. To do so, we increased the level of acetate in SPO from 0.3 to 1.0% w/v, and the medium was supplemented with amino acids and adenine (see *Materials and Methods* for details). Cells started meiotic divisions slightly earlier when using the supplemented SPO instead of the regular SPO (Figure 2F). Hence, we decided to use the supplemented SPO for the experiments described in the remainder of the manuscript.

### Induction of IME1 is sufficient to induce gametogenesis synchronously

**Figure 3:**
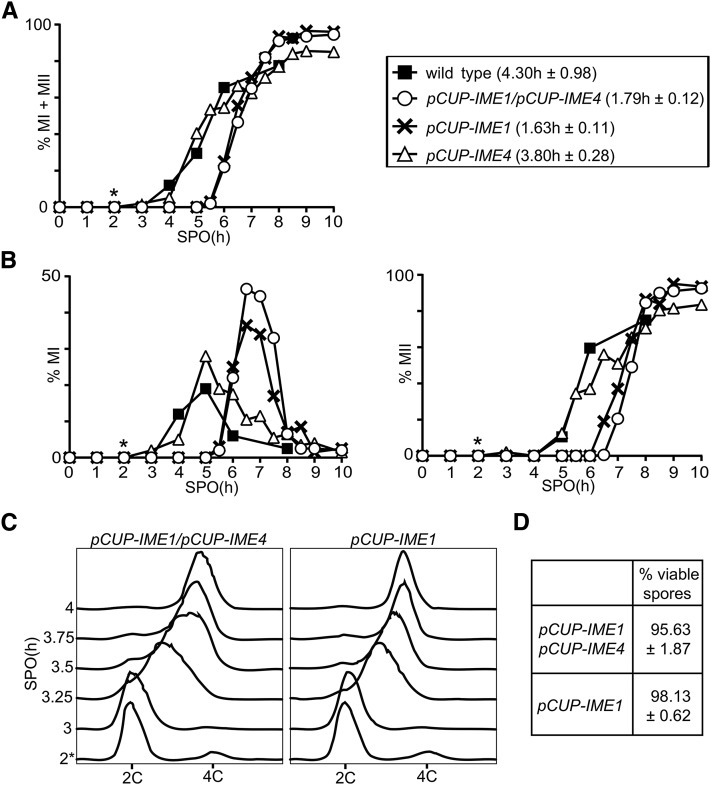
Induction of *IME1* is sufficient to induce gametogenesis synchronously. (A) Kinetics of meiotic divisions in wild-type cells (FW1511), cells harboring *IME1* and *IME4* fused to the *CUP1* promoter (*pCUP-IME1/pCUP-IME4*) (FW1810), *pCUP-IME1* (FW2444), or *pCUP-IME4* (FW2480). Cells were grown overnight in YPD, and shifted to SPO (1.0% w/v potassium acetate supplemented with adenine, uracil, and amino acids); 50 µM copper (II) sulfate was added 2 hr after the cells were transferred to SPO. Samples were taken at the indicated time point, fixed in ethanol, nuclei were stained with DAPI, and DAPI masses were counted. Cells that harbored two, three, or four DAPI masses were classified as cells undergoing meiosis I or meiosis II (% MI + MII). For each time point, at least 200 cells were counted. We also computed the time or period taken for 75% of the cells to complete meiotic divisions (see *Materials and Methods* for details). This number is displayed in brackets next to the legend, and represents the mean number of hours followed by the SEM of three independent experiments. (B) Similar to A except that the percentages of bi- (left panel), tri-, and tetra-nucleate (right panel) cells are shown. (C) Flow cytometry analysis of DNA content of *pCUP-IME1*/*pCUP-IME4* (FW1810) and the *pCUP-IME1* (FW2444) cells that were induced to sporulate as described in (A). Samples were taken at indicated time points, fixed, and DNA content was measured by propidium iodide staining; 50 µM copper (II) sulfate was added 2 hr after the cells were transferred to SPO. At least 50,000 cells were analyzed at each time point. (D) Spore viability of the *pCUP-IME1*/*pCUP-IME4* (FW1810) and the *pCUP-IME1* (FW2444) cells that were induced to sporulate as described in (A). Tetrads were collected 24 hr after induction, dissected, and assayed for viability (*n* = 160 spores). The average result and the SEM of three independent experiments is shown. *, time of induction.

Both *IME1* and *IME4* have been shown to promote entry into sporulation (van Werven and Amon 2011). Whereas *ime4* deletion mutants do not sporulate in certain strain backgrounds, but do in others, *IME1* is essential for sporulation in *Saccharomyces cerevisiae* (Kassir *et al.* 1988; Shah and Clancy 1992; Hongay *et al.* 2006). *IME4* has been implicated to positively regulate *IME1* expression (Shah and Clancy 1992). We hypothesized that, if *IME1* and *IME4* regulate each other, then perhaps synchronous sporulation should require controlled expression of either *IME1* or *IME4* alone. To test this, we measured the period taken to complete meiotic divisions when *IME1*, *IME4*, or both, were induced from the *CUP1* promoter. One-way ANOVA showed that there was a statistically significant difference between the group means, *F*(3,8) = 6.97, *P* = 0.0127. We found that the kinetics of meiotic divisions of cells that express *pCUP-IME4* only was comparable to wild-type control cells (Figure 3A). In contrast, the kinetics of meiotic division in cells harboring *pCUP-IME1* alone, or both *pCUP-IME1* and *pCUP-IME4*, significantly improved when compared to the wild-type control, which was confirmed by a *post hoc* Dunnett’s test (cf. 1.63 hr and 1.79–4.30 hr, *P* = 0.0166 and *P* = 0.0223, respectively). The results were similar when we examined the kinetics of meiosis I and meiosis II separately (Figure 3B). To investigate more closely whether induction of *pCUP-IME1* alone is sufficient for synchronous sporulation, we also monitored the kinetics of premeiotic DNA replication (Figure 3C). We found that cells harboring either *pCUP-IME1* or *pCUP-IME1/pCUP-IME4*, underwent premeiotic DNA replication synchronously within ∼45 min, and gave rise to viable spores (Figure 3D). In conclusion, temporal expression of *IME1* alone is sufficient to induce synchronous sporulation.

### IME1 directly regulates the expression of IME4

**Figure 4:**
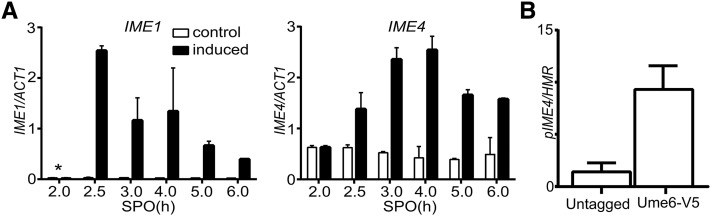
*IME1* directly regulates the expression of *IME4*. (A) Induction of *IME1* promotes *IME4* expression. Diploid cells harboring *pCUP-IME1* (FW2444) were transferred to SPO. Cells were either untreated (control) or treated with copper (II) sulfate (induced), and samples were collected at different time points. Total RNA was isolated, reverse transcribed, and *IME1* (left panel) or *IME4* (right panel) mRNA levels were measured by quantitative PCR. To quantify *IME4* levels, primers specific for the *IME4* and *ACT1* sense strand were used in the reverse transcription reaction. Signals were normalized to *ACT1* levels. The values and error bars represent two independent experiments. (B) Ume6 binds to the promoter of *IME4*. Diploid cells harboring Ume6 tagged with the V5 epitope (FW1208) and a wild-type control strain (FW1511) were grown in YPD to saturation. Cells were fixed with formaldehyde, and cells were processed for ChIP analyses (see *Materials and Methods* for details). DNA fragments specific to the *IME4* promoter (*pIME4*) were amplified and quantified by qPCR. Signals were normalized to the *HMR* locus. The error bars represent the SE of at least three experiments. *, time of induction of *IME1*—2 hr after the cells were transferred to SPO.

The observation that temporal expression of *IME1*, but not of *IME4*, generates a high degree of synchrony during DNA replication and meiotic divisions, prompted us to revisit how the two genes regulate each other. We hypothesized that *IME1* directly or indirectly regulates *IME4* expression. To examine this possibility, we measured *IME4* transcript levels in cells harboring *pCUP-IME1* in the presence or absence of copper (II) sulfate. As expected, *IME1* transcript levels increased when copper ions were added to the SPO medium (Figure 4A). Since *IME4* is also regulated by an antisense transcript, we specifically quantified *IME4* sense mRNA using a transcript-specific primer in the reverse transcription reaction (Hongay *et al.* 2006; Gelfand *et al.* 2011). We found that *IME4* transcript levels significantly increased when *IME1* was induced, suggesting that Ime1 stimulates *IME4* transcription (Figure 4B). Data from a genome-wide study indicated that *IME4* is regulated directly by the repressor Ume6 (Williams *et al.* 2002). During early sporulation, Ime1 interacts with Ume6 to form a transcription-activating complex for the expression of early meiotic genes (Bowdish *et al.* 1995; Rubin-Bejerano *et al.* 1996). To test whether Ume6 indeed binds the *IME4* promoter, we identified the canonical URS1 motif (TAGGCGGC) sequence at −234 bp upstream in the *IME4* promoter. More importantly, we found that Ume6 was bound directly to the *IME4* promoter, as shown by ChIP (Figure 4C). In conclusion, our results show that *IME1* directly regulates the expression of *IME4*, explaining why *IME1* can single-handedly induce synchronous sporulation. These results also suggest that *IME1* and *IME4* act in a positive feedback loop to stimulate the expression of each other.

### Combining the pCUP-IME1 system with another synchronization method

**Figure 5:**
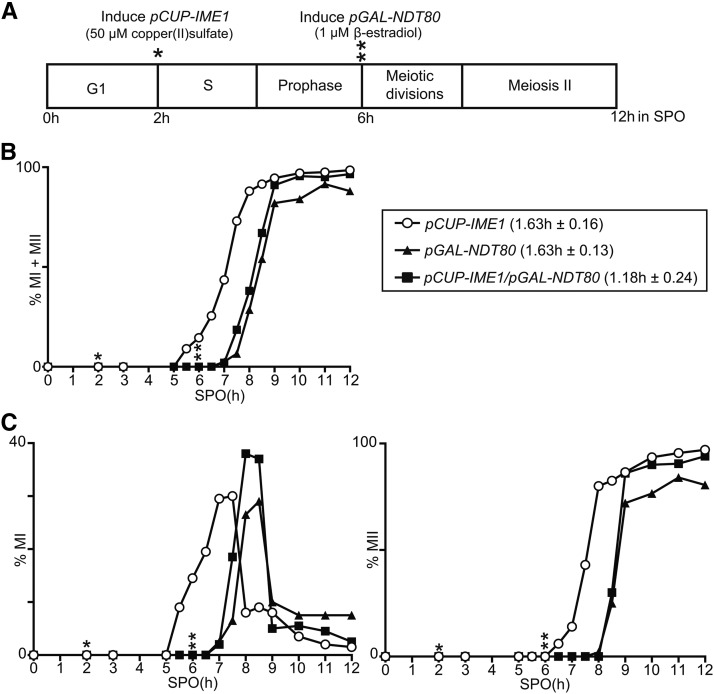
*pCUP-IME1* system can be combined with the *pGAL-NDT80* system to improve synchrony of sporulation. (A) Scheme of experimental setup. The diploid cells harboring *IME1* fused to *CUP* promoter (FW2444), *NDT80* expressed from the *GAL* promoter together with Gal4 fused to the estrogen receptor (*GAL4.ER pGAL-NDT80*) (FW1541) or a strain expressing both (*pCUP-IME1* and *GAL4.ER pGAL-NDT80*) (FW2795) were grown in YPD overnight. Cells harboring *GAL4.ER GAL-NDT80* (FW1541) were transferred to presporulation medium (BYTA). Subsequently, cells were pelleted by centrifugation, washed with sterile water and resuspended to a final OD_600_ of 2.5 in SPO; 50 µM copper (II) sulfate was added 2 hr after the cells were transferred to SPO, and 1 µM β-estradiol was added 6 hr after transfer to SPO. (B) Kinetics of meiotic divisions in strains, using procedures described in (A). Samples were taken at the indicated time points, fixed in ethanol, nuclei were stained with DAPI, and DAPI masses were counted. Cells that harbored two, three, or four DAPI masses were classified as cells undergoing meiosis I or meiosis II (% MI + MII). For each time point, at least 200 cells were counted. We also computed the time or period taken for 75% of the cells to complete meiotic divisions (see *Materials and Methods* for details). This number is displayed in brackets next to the legend, and represents the mean number of hours followed by the SEM of three independent experiments. (C) Similar to B except that the percentages of bi- (left panel), or tri- and tetra-nucleate (right panel) cells (*n* = 200 cells) of strains described in (A) were determined. *, time of *IME1* induction; **, time of *NDT80* induction.

Different genetic approaches have been used to synchronize cells at different stages of sporulation (Benjamin *et al.* 2003; Wan *et al.* 2006; Carlile and Amon 2008). One approach makes use of controlled expression of the transcription factor *NDT80*, and, as a result, cells undergo meiotic divisions synchronously (Benjamin *et al.* 2003; Carlile and Amon 2008). The Ndt80 transcription factor promotes the expression of numerous genes that regulate meiotic divisions, also known as middle genes (Xu *et al.* 1995; Chu *et al.* 1998). Effective induction of *NDT80* is achieved by controlling its expression from the *GAL1* promoter (*pGAL-NDT80*) and the transcription factor *GAL4-ER*, consisting of the Gal4 DNA binding domain fused to the estrogen receptor binding domain. In the presence of β-estradiol, *pGAL-NDT80* cells induce *NDT80*, and exit from pachytene arrest to undergo meiotic divisions (Benjamin *et al.* 2003; Carlile and Amon 2008). The *pGAL-NDT80* system specifically synchronizes meiotic divisions during gametogenesis, but, unlike the *pCUP-IME1* system, this method does not synchronize the events prior to meiotic chromosome segregation. To examine whether it is possible to combine the *pCUP-IME1* system with the *pGAL-NDT80* system, we generated a diploid strain with both synchronization systems. The early and middle stages of sporulation were initiated by *IME1* at 2 hr, and *NDT80* at 6 hr, after shifting cells to SPO, respectively (Figure 5A). We found that the *pCUP-IME1* and *pGAL-NDT80* cells had a similar degree of synchrony of meiotic divisions (Figure 5B). The *pCUP-IME1/pGAL-NDT80* strain showed a minor improvement in synchrony, which was not statistically significant (*P* > 0.05), when compared to cells expressing *pCUP-IME1* or *pGAL-NDT80* alone (1.18 hr compared to 1.63 and 1.63 hr) (Figure 5B). We observed a similar trend when we examined meiosis I and meiosis II divisions separately, showing that the *pCUP-IME1* or *pGAL-NDT80* systems can be combined (Figure 5C). With the *pCUP-IME1/pGAL-NDT80* system, we can synchronize cells at the level of premeiotic DNA replication until completion of meiotic divisions, with the added advantage of being able to control entry into the early and middle stages of gametogenesis.

## Discussion

Here, we demonstrate that temporal expression of a single gene, *IME1*, induces sporulation synchronously in budding yeast. Our approach requires neither preculturing in acetate-containing medium nor expressing *IME4* from a heterologous promoter. The system described here can be combined easily with other synchronization methods, and will be of use for studying specific stages of sporulation, or the complete sporulation program.

Our data indicate that induction of synchronous sporulation requires a specified timing of *IME1* induction. Interestingly, in wild-type cells, the *IME1* promoter, like in synchronous sporulation, is active at 2 hr after shifting to sporulation medium (Inai *et al.* 2007; Nachman *et al.* 2007). Given that nutrient availability is an important trigger for *IME1* and sporulation, perhaps nutrients also control timing of sporulation after *IME1* induction (Jambhekar and Amon 2008; van Werven and Amon 2011) . Inducing *IME1* too late could affect sporulation, because cells have been starved for prolonged times, whereas inducing *IME1* too early does not result in optimal sporulation because cells are not ready. In line with this hypothesis, in a recent report we showed that a certain level of nutrient-sensing target of rapamycin complex (TORC1) activity is needed for sporulation (Weidberg *et al.* 2016). Too much or too little TORC1 affects sporulation negatively. Perhaps, TORC1 activity is most optimal for synchronous sporulation at 2 hr in SPO. Another explanation is that downstream targets of *IME1* are not properly activated because the meiosis promoting kinases Rim11 and Rim15 are not active (Bowdish *et al.* 1994; Rubin-Bejerano *et al.* 1996; Vidan and Mitchell 1997; Pedruzzi *et al.* 2003; Sarkar *et al.* 2014). This can explain why inducing *IME1* too early did not result in synchronous sporulation, but cannot explain the reduced synchrony when *IME1* is induced too late. More work is needed to pinpoint why timing of *IME1* expression is critical for synchronous sporulation.

Our work sheds light on how *IME1* and *IME4* regulate each other in wild-type cells. It has been shown that *ime4* mutant cells have diminished levels of *IME1* (Shah and Clancy 1992). In addition, one report showed that *IME1* transcripts contain the m6A modification, suggesting that Ime4 controls *IME1* directly (Bodi *et al.* 2010). However, genome-wide sequencing of m6A did not identify the modification in *IME1* (Schwartz *et al.* 2013). In this study, we demonstrate that *IME1* can also directly regulate *IME4* expression. Our data show that *IME4* levels increased when *IME1* was induced. We also find that the Ume6 repressor is bound to the promoter of *IME4*. Others have shown that *IME4* transcripts accumulate later in sporulation than *IME1*, which also supports the idea that *IME4* can be downstream of *IME1* (Primig *et al.* 2000; Nachman *et al.* 2007). We propose that *IME1* and *IME4* can positively regulate each other. The advantage of such dynamic regulation is that it allows for rapid accumulation of both transcripts when cells are ready to undergo sporulation.

Several other approaches have been used to synchronize cells throughout, or at specific stages of, sporulation. First, the sporulation-proficient SK1 strain background can undergo sporulation efficiently, and with some degree of synchrony, when specific growth conditions are adopted (Kane and Roth 1974; Falk *et al.* 2010; Borner and Cha 2015). We show that the *pCUP-IME1* strain reaches a much better synchrony in comparison to wild-type SK1. Second, mutations that cause cells to arrest at specific stages of gametogenesis are also used to synchronize cell populations. For example, *ime2* mutants arrest prior to DNA replication, whereas *ndt80* mutants arrest in meiotic prophase I (Xu *et al.* 1995; Dirick *et al.* 1998). Although these approaches are useful for studying specific stages, they have several limitations. For example, only one stage per mutant can be studied, and not all stages can be arrested. Third, there are other “block and release” genetic approaches that reversibly arrest, and then synchronize, cell populations in certain stages of sporulation. For example, the *pGAL-NDT80* system synchronizes meiotic divisions (see previous section) (Carlile and Amon 2008). Another example of stage-specific synchronization is the analog-sensitive allele of *CDC7* (*cdc7-as3*), which is used to arrest cells following premeiotic S-phase, and synchronizes cells through homologous recombination and meiosis I (Wan *et al.* 2006; Lo *et al.* 2008; Wan *et al.* 2008). However, these approaches synchronize cells only for a selective part of gametogenesis. Our data show that the *pCUP-IME1* system can achieve a high degree of synchrony during premeiotic DNA replication and meiotic divisions. The *pCUP-IME1* system also dispenses with the need to use presporulation medium, which shortens the procedure to 2 d. The *pCUP1-IME1* system can be used alone, or combined with other synchronization systems, as a tool to profile gene expression or protein production patterns throughout gametogenesis. Finally, the high degree of synchrony achieved by our method will be useful in dissecting the different stages in finer detail, or to study temporal coordination and regulation of events during gametogenesis.

## Supplementary Material

Supplemental Material
