# Supplementary material for: Temporal Expression of a Master Regulator Drives Synchronous Sporulation in Budding Yeast
Source: G3 (Bethesda). 2016 Sep 6;6(11):3553–60. doi: 10.1534/g3.116.034983 (PMC5100854; doi:10.1534/g3.116.034983)
Supplement: Supplemental Material [file supp_6_11_3553__index.html]

Temporal Expression of a Master Regulator Drives Synchronous Sporulation in Budding Yeast — Supplemental Material 

# Temporal Expression of a Master Regulator Drives Synchronous Sporulation in Budding Yeast

## Supplemental Material for Chia , *et al*, 2016

**Files in this Data Supplement:**

- Table S1 - Genotype of strains used in this study. (.pdf, 209 KB)
