# Supplementary material for: Temporal Expression of a Master Regulator Drives Synchronous Sporulation in Budding Yeast
Source: G3 (Bethesda). 2016 Sep 6;6(11):3553–60. doi: 10.1534/g3.116.034983 (PMC5100854; doi:10.1534/g3.116.034983)
Supplement: Supplemental Material [file supp_g3.116.034983_TableS1.pdf]

Table S1. Genotype of strains used in this study.

Strain	*Genotype*
FW1208	*MAT***a,** *ho::LYS2, ura3, leu2::hisG, his3::hisG, trp1::hisG, UME6-3V5::HIS3*<br>*MATα***,** *ho::LYS2, ura3, leu2::hisG, his3::hisG, trp1::hisG, UME6-3V5::HIS3*
FW1509	*MAT***a***, ho::LYS2, lys2, ura3, leu2::hisG, his3::hisG, trp1::hisG*
FW1510	*MATα, ho::LYS2, lys2, ura3, leu2::hisG, his3::hisG, trp1::hisG*
FW1511	*MAT***a***, ho::LYS2, lys2, ura3, leu2::hisG, his3::hisG, trp1::hisG*<br>*MATα, ho::LYS2, lys2, ura3, leu2::hisG, his3::hisG, trp1::hisG*
FW1541	*MAT***a***, ho::LYS2, lys2, ura3, leu2::hisG, his3::hisG, trp1::hisG*<br>*ndt80::pGAL-NDT80::TRP1, ura3::pGPD1-GAL4(848).ER::URA3*<br>*MATα, ho::LYS2, lys2, ura3, leu2::hisG, his3::hisG, trp1::hisG*<br>*ndt80::pGAL-NDT80::TRP1, ura3::pGPD1-GAL4(848).ER::URA3*
FW1810	*MAT***a***, ho::LYS2, lys2, ura3, leu2::hisG, his3::hisG, trp1::hisG, irt1::pCUP-3HA-IME1::KANMX, ime4::pCUP-3HA-IME4::KANMX*<br>*MATα, ho::LYS2, lys2, ura3, leu2::hisG, his3::hisG, trp1::hisG, irt1::pCUP-3HA-IME1::KANMX, ime4::pCUP-3HA-IME4::KANMX*
FW2444	*MAT***a***, ho::LYS2, lys2, ura3, leu2::hisG, his3::hisG, trp1::hisG, irt1::pCUP-3HA-IME1::KANMX*<br>*MATα, ho::LYS2, lys2, ura3, leu2::hisG, his3::hisG, trp1::hisG, irt1::pCUP-3HA-IME1::KANMX*
FW2480	*MAT***a***, ho::LYS2, lys2, ura3, leu2::hisG, his3::hisG, trp1::hisG, ime4::pCUP-3HA-IME4::KANMX*<br>*MATα, ho::LYS2, lys2, ura3, leu2::hisG, his3::hisG, trp1::hisG, ime4::pCUP-3HA-IME4::KANMX*
FW2795	*MAT***a***, ho::LYS2, lys2, ura3, leu2::hisG, his3::hisG, trp1::hisG, irt1::pCUP-3HA-IME1::HPHMX , ndt80::pGAL-NDT80::TRP1, ura3::pGPD1-GAL4(848).ER::URA3*<br>*MATα, ho::LYS2, lys2, ura3, leu2::hisG, his3::hisG, trp1::hisG, irt1::pCUP-3HA-IME1::HPHMX , ndt80::pGAL-NDT80::TRP1, ura3::pGPD1-GAL4(848).ER::URA3*
